# *Limosilactobacillus reuteri* SXDT-32-derived shikimic acid protects against colonic inflammation in piglets by inhibiting the PI3K-Akt pathway

**DOI:** 10.1186/s40104-025-01221-w

**Published:** 2025-06-13

**Authors:** Ying Chen, Chengzeng Luo, Zhaohan Zhan, Shuo Liu, Chunran Teng, Ruixiao Mao, Shunfen Zhang, Xunbozan Zhang, Qingshi Meng, Ruqing Zhong, Liang Chen, Hongfu Zhang

**Affiliations:** 1 State Key Laboratory of Animal Nutrition and Feeding, Institute of Animal Sciences, Chinese Academy of Agricultural Sciences, Beijing, 100193 China; 2 College of Animal Science, Shanxi Agricultural University, Jinzhong, 030801 China; 3 College of Animal Science and Technology, Qingdao Agricultural University, Qingdao, 266109 China

**Keywords:** Intestinal inflammation, *L. reuteri* SXDT-32, PI3K-Akt pathway, Shikimic acid

## Abstract

**Background:**

Colitis caused by bacterial infection is a major global health challenge. Unfortunately, current treatment options are limited. We previously disclosed that *L. reuteri* SXDT-32 was enriched in the feces of an ancient diarrhea-resistant pig breed (Mashen pig) in China over 2500 years old. As diarrhea is often closely associated with intestinal inflammation, *L. reuteri* SXDT-32 was identified as a potential beneficial bacterium to prevent intestinal inflammation. However, the precise mechanisms involved remained unclear.

**Results:**

Our tests showed that *L. reuteri* SXDT-32 alleviated colonic damage induced by pathogenic *E. coli* SKLAN202302 in weaned pigs by enhancing barrier integrity and inhibiting inflammation. The transcriptomics revealed that *L. reuteri* SXDT-32 protected against inflammatory injury by inhibiting the PI3K-AKT signaling pathway. Metabolite analysis indicated that the content of shikimic acid (SA) was substantially elevated in the colonic mucosa of *L. reuteri* SXDT-32-fed piglets (*P* < 0.05). In addition, Liquid Chromatography-Mass Spectrometer (LC-MS) analysis showed significant increases in SA content in both the colonic chyme of *L. reuteri* SXDT-32-fed piglets and the supernatant of in vitro grown cultures of *L. reuteri* SXDT-32 (*P* < 0.05). Polymerase chain reaction (PCR) analysis identified gene *aroE* from *L. reuteri* SXDT-32, which is a key gene directly linked to SA synthesis, and elevated shikimate dehydrogenase (SD, encoded by *aroE*) was also detected in both *L. reuteri* SXDT-32 and the colonic mucosa of piglets fed *L. reuteri* SXDT-32 (*P* < 0.01). In vitro Caco-2 cell experiments demonstrated that SA, *L. reuteri* SXDT-32, and the supernatant from in vitro grown cultures of *L. reuteri* SXDT-32 exhibited comparable inhibitory effects on the PI3K-Akt pathway to those of the PI3K inhibitor LY294002.

**Conclusions:**

*L. reuteri* SXDT-32 alleviated intestinal inflammation in piglets by producing SA that inhibits the PI3K-Akt pathway. This study provides an innovative approach for the treatment and prevention of colitis caused by bacterial infection.

**Supplementary Information:**

The online version contains supplementary material available at 10.1186/s40104-025-01221-w.

## Introduction

Colitis caused by bacterial infection, such as enteropathogenic *Escherichia coli* (EPEC) and enterohemorrhagic *Escherichia coli* (EHEC), is a major challenge for people all over the world [1, 2]. However, current treatment options for colitis caused by bacterial infection are limited and not suitable for long-term use due to side effects [3]. Probiotics have shown potential in alleviating colitis caused by bacterial infection, attributed to their anti-inflammatory effects, enhancement of intestinal barrier function, and modulation of immune responses [4]. An in-depth understanding of potential protective effects of probiotics against intestinal inflammation may lead to better treatments for colitis caused by bacterial infection.

Native pig breeds exhibit greater resistance to diarrhea than commercial breeds [5, 6]. Emerging evidence emphasizes its strong association with the intestinal microbiota [7]. Presumably, native pigs may contain specific anti-diarrheal beneficial bacteria within their intestines. For example, previous studies have shown that native Congjiang miniature (CM) piglets exhibit greater resistance to diarrhea compared to commercial pigs. This increased resistance is primarily attributed to the significant enrichment of *Lactobacillus gasseri* LA39 and *Lactobacillus frumenti* in their intestines [8]. Similarly, our earlier research revealed that native Mashen piglets were also more resistant to early weaning stress-induced diarrhea compared to commercial Duroc × Landrace × Yorkshire (DLY) piglets. Research also revealed that *L. reuteri* SXDT-32, a bacterium with good probiotic properties was significantly enriched in the feces of Mashen piglets. Thus, we hypothesized that the reduced incidence of diarrhea in Mashen piglets may be associated with the enrichment of *L. reuteri* SXDT-32 in their intestines. Given the tight link between diarrhea and intestinal inflammation [9], *L. reuteri* SXDT-32 may relieve diarrhea by alleviating intestinal inflammation. However, the exact role and mechanism of the *L. reuteri* SXDT-32 remained unclear.

*L. reuteri*, a well-known probiotic, is essential for sustaining the balance of gut microbiota, restraining the growth of intestinal disease-causing bacteria, regulating barrier integrity and immune function [10–12]. Therefore, it is widely used in the treatment of infantile rotavirus diarrhea, infantile bowel disease and ulcerative colitis [13–16]. *L. reuteri* influences bowel health through several mechanisms, such as modulating signaling pathways, generating antimicrobial substances such as lactic acid, hydrogen peroxide, and preventing infections by competing with pathogens for receptors and binding sites on the intestinal mucosa [17–21]. Notably, a majority of studies indicated that *L. reuteri* is capable of relieving intestinal inflammation through the generation of specific metabolites. These metabolites have been found to play a major role in alleviating intestinal inflammation [22, 23]. For example, histamine from *L. reuteri* is able to relieve colitis. It achieves this by inhibiting the pro-inflammatory type 1 histamine receptors H1R and by stimulating the anti-inflammatory type 2 histamine receptors H2R [24]. Similarly, another study suggested that the indole-3-aldehydes from *L. reuteri* can contribute to the activation of the AhR-IL-22 axis and the maintenance of intestinal microbial balance [25]. Therefore, we hypothesized that *L. reuteri* SXDT-32 was able to relieve intestinal inflammation by producing critical anti-inflammatory metabolites. Our research utilized a piglet model of intestinal inflammation induced by *E. coli* SKLAN202302 to explore the action mechanism of *L. reuteri* SXDT-32 in protecting piglets against intestinal inflammation. The findings of this research emphasized the potential application of *L. reuteri* SXDT-32 in the prevention of other intestinal inflammatory diseases in the future.

## Materials and methods

### Bacterial strains

*L. reuteri* SXDT-32 strain, isolated from Mashan pig feces by our lab, was preserved in the China General Microbiological Culture Collection Center (CGMCC, No. 24727) and grown in de Man, Rogosa and Sharpe (MRS) broth (AOBOX, 02-291; Beijing Aoboxing Biology Technology Co., Ltd., China) at 37 °C, 180 r/min for 12 h prior to the experiment.

*E. coli* SKLAN202302 strain, isolated from colonic chyme of diarrheic Duroc × Landrace × Yorkshire piglets by our lab, was preserved in the China General Microbiological Culture Collection Center (CGMCC, No. 26420) and grown in MacConkey broth (AOBOX, 02-369) at 37 °C, 180 r/min for 12 h prior to the experiment.

### Animals and experimental treatment

Twenty-four Duroc × Landrace × Yorkshire (DLY) weaned piglets (21 d, 6.15 ± 0.10 kg) were randomly distributed into three equal groups (eight replicates per group, one pig per replicate, balanced for body weight and sex): Control group (Con), *E. coli* group (E.c) and *L. reuteri* + *E. coli* group (L.r + E.c). The basal diet was fed to both the Con and E.c groups, while the L.r + E.c group received the same diet with the addition of *L. reuteri* SXDT-32 (1.0 × 10^8^ CFU/d per pig). On d 7, d 10, and d 15, 1 mL, 2 mL, and 3 mL of *E. coli* SKLAN202302 (1.0 × 10^8^ CFU/mL) were intraperitoneally injected into pigs in the E.c and L.r + E.c groups, respectively, whereas the Con group received the same volumes of 0.9% saline. The experimental period was 18 d. During the experiment, all piglets were housed indoors with temperature controlled at 24–26 °C and relative humidity at 50%–70%. They had unrestricted access to feed and water. The ingredient composition and nutrient level of the basal diet were shown in Table S1. Body weights were measured on d 0, d 7 and d 17. After 18 d of trial, all piglets were humanely slaughtered, after which colonic mucosa and colonic chyme samples were collected, flash-frozen in liquid nitrogen, and stored in a −80 °C freezer for subsequent analysis. Fresh colon tissues were preserved in 4% paraformaldehyde for Hematoxylin-eosin (HE) staining, Alcian Blue-Periodic Acid-Schiff (AB-PAS) and Fluorescence In Situ Hybridization (FISH).

An additional 24 DLY weaned piglets were grouped, treated and housed following the same methods as described above, but they were not sacrificed. After 18 d, no further treatments were applied to any of the groups. Instead, they were only provided with the basal diet. Fecal samples from the piglets were collected on d 9, d 17, d 24, and d 31 of the experiment. These samples were subsequently used to quantify the number of lactobacilli in the feces.

### Enumeration of fecal lactobacilli

Fecal samples (1 g) were collected from each piglet on d 9, d 17, d 24 and d 31. These samples were then transferred to an aseptic test tube containing 9 mL of sterile distilled water and subjected to a serial ten-fold dilution ranging from 10^–1^ to 10^–6^. Subsequently, 20 μL of each diluted sample was plated onto MRS agar (BD-Pharmingen, 288210) and incubated at 37 °C for 24 h.

### Fluorescence In Situ Hybridization (FISH)

Three colonic tissue samples were randomly selected from each group. After fixation in 4% paraformaldehyde overnight, the samples were paraffin-embedded and sectioned into 5-μm-thick slices. The sections were hybridized in a solution that contained 0.01% SDS, 20 mmol/L Tris-HCl (pH 7.3), and 0.9 mol/L NaCl. A Cy3-labeled probe (5'-TTCACATCAGACCTAAGCAACCGCCTGCGCTCGC-3') was designed based on the 16S rRNA gene sequence of *L. reuteri* SXDT-32. The hybridization buffer was added to the tissue sections, heated, and then combined with 8 ng/μL of the DNA probe. The sections were washed with 2× SSC at 37 °C for 10 min, followed by washing with 1× SSC at 37 °C for 2× 5 min, and finally washed with 0.5× SSC at ambient temperature for 10 min. The sections were incubated with a DAPI staining solution at a concentration of 2 µg/mL in the dark for 8 min. Following the rinse, the sections were mounted in fluorescence-anti-quenching mounting medium. Microscopic observation was performed using an Olympus fluorescence microscope (Olympus Corporation, Tokyo, Japan). The fluorescence intensity was quantified using ImageJ 1.8.0 software.

### Histological analysis

Five-μm thick sections were prepared from paraffin-embedded colon tissue fixed in 4% paraformaldehyde (Solarbio, P1110-500). The sections were stained with Hematoxylin-eosin (HE). Images were captured with a DM300 microscope (Leica, Germany). Histological scores were quantified by two blinded researchers according to the following histological grading criteria for colitis. Grade 0 indicates no damage. Grade 1 represents local congestion without ulcer. Grade 2 is characterized by a linear ulcer without inflammation. Grade 3 shows a linear ulcer with mild inflammation. Grade 4 refers to multiple ulcers larger than 1 cm. For Grades 5-8, when the damage is greater than 2 cm, the score increases by 1 for each additional centimeter [26].

For goblet cell detection, 5-μm thick colon tissue sections were stained with Alcian Blue-Periodic Acid-Schiff (AB-PAS) according to the instructions of the AB-PAS Stain Kit (Solarbio, G1285). Images were recorded using a DM300 microscope (Leica, Germany). Goblet cell counts were determined with ImageJ 1.8.0 software.

### RNA extraction and quantitative real-time polymerase chain reaction (qRT-PCR)

Total RNA was extracted from colonic mucosa samples according to the instructions of the Trizol reagent. RNA concentrations were measured employing a NanoDrop Lite spectrophotometer (Thermo Fisher, MA, USA). Before the process of reverse transcription, genomic DNA impurity was eliminated according to the instructions of the gDNA Eraser from the PrimeScript^TM^ RT reagent kit (Takara, RR047A). QRT-PCR was performed according to the instructions of the SYBR^®^ Premix Ex Taq^TM^ kit (Takara, RR420A). The relative expression levels of genes were calculated with the 2^−ΔΔCt^ method. The reference gene used in these computations was *ACTB*. Primer design (Table S2) was performed using the NCBI primer design tool (http://www.ncbi.nlm.nih.gov/tools/primer-blast/).

### Western blot

For the piglet experiment, 4 colonic mucosa samples were randomly selected from each group. Similarly, quadruplicate biological replicates (*n* = 4 per group) were established for the Caco-2 cell experiments. All samples were lysed using RIPA buffer for protein extraction. The SDS-PAGE technique was employed to resolve total proteins, which were then blotted onto 0.45-μm PVDF membranes supplied by Millipore, USA. After blocking the membranes for 2 h in a solution containing 5% skim milk, they were incubated with the primary antibodies at 4 °C overnight. Following the incubation with primary antibodies, the membranes were then exposed to secondary antibodies for 1 h under ambient conditions. Ultimately, protein detection was facilitated by an ECL kit (C081909, YangGuangBio, Beijing, China), and subsequent visualization was performed with a Chemi XRS system (Bio-Rad, California, USA). The ImageJ 1.8.0 software was used to quantify the band intensity. Primary antibodies were obtained from different suppliers: OCLN (Bioss, bs-10011R, 1:2,000 dilution), CLDN1 (Bioss, bs-0790R, 1:2,000 dilution), IL-22 (Abcam, #ab193813, 1:2,000 dilution), IL-1β (Bioss, bs-0812R, 1:2,000 dilution), p-PI3Kp85 (Affinity, AF3241, 1:2,000 dilution), PI3K (Proteintech, 60225-1-lg, 1:2,000 dilution), p-Akt (Affinity, AF0016, 1:2,000 dilution), Akt (Proteintech, 10176-2-AP, 1:2,000 dilution), and β-actin (Servicebio, ZB15001-HRP-100, 1:2,000 dilution). Secondary antibodies were purchased from abcam (ab6721, 1:5,000 dilution) and abcam (ab205719, 1:5,000 dilution). Primary antibody dilution buffer was obtained from YangGuangBio (C210202). Secondary antibody dilution buffer was purchased from YangGuangBio (C210201).

### Transcriptome analysis

The phenol-chloroform extraction technique was used to extract total RNA from colonic mucosa samples. During the construction of the sequencing library, the extracted RNA samples underwent quality control. High-quality RNA samples were required to meet the following criteria: OD_260/280_ = 1.8–2.2, OD_260/230_ ≥ 1.0, RIN ≥ 6.5, 28 S:18 S ≥ 1.0, and the amount was > 10 μg. Majorbio Company (Shanghai, China) carried out the RNA sequencing. The raw data were deposited in the NCBI Sequence Read Archive (PRJNA1202552). Data were analyzed using Majorbio ISanger Cloud Platform (https://cloud.majorbio.com/page/project/p.html). Genes with |log_2_(fold change)| ≥ 1 and *P* value < 0.05 were considered differentially expressed genes (DEGs). The DEGs were subjected to functional annotation and pathway analysis using the Gene Ontology (GO) and the Kyoto Encyclopedia of Genes and Genomes (KEGG) databases.

### Metabolomics analysis

Colonic mucosa samples (50 mg each) or freeze-dried *L. reuteri* SXDT-32 supernatants (20 mg each) were precisely weighed. Each sample was then treated with 400 µL of an internal standard solution containing 0.02 mg/mL L-2-chlorophenylalanine in 80% methanol. The mixtures were incubated at −20 °C for 30 min to allow protein precipitation and then vortexed for 30 s to ensure thorough mixing. Subsequently, the samples underwent centrifugation at 13,000 r/min for 15 min at 4 °C. The resulting supernatant was then transferred to new vials. The chromatographic separation utilized a Thermo UHPLC system fitted with an ACQUITY UPLC HSS T3 column (100 mm × 2.1 mm, 1.8 µm). After the samples were prepared, they were analyzed using a Thermo UHPLC-Q Extractive HF-X mass spectrometer, equipped with an electrospray ionization source. The raw data were loaded into Progenesis QI 3.0 software for peak recognition, alignment, and standardization. Metabolites exhibiting Variable Importance in the Projection (VIP) > 1 and *P* < 0.05 were recognized as differentially abundant.

### Amplification of the *aroE* gene of *L. reuteri* SXDT-32 and assay for the activity of shikimate dehydrogenase (SD)

According to the instructions of the Steady Pure Bacterial Genomic DNA Extraction Kit (Takara, 9763), DNA of *L. reuteri* SXDT-32 was extracted. The *aroE* gene (accession number: NP_417740.1; amplified sequence: 201 bp) was amplified according to the instructions of the TaKaRa Taq^TM^ (Takara, R001A), using primers F: 5'-CTGGACTGTGCGGTGACAATAAC-3' and R: 5'-GCCTGGATGAATGAGCGATGAC-3'. Subsequently, a 1% agarose gel was prepared, and the amplified products were electrophoresed to visualize the amplified DNA products.

According to the instructions of Shikimate Dehydrogenase Activity Assay Kit (BOXBIO, AKAM018M), the activity of SD in the *L. reuteri* SXDT-32 and the colonic mucosa was detected.

### Liquid Chromatography-Mass Spectrometer (LC-MS) analysis of SA

SA was extracted using distilled water from both *L. reuteri* SXDT-32 supernatant and colonic chyme. Samples were processed at 70 Hz for 90 s using a Scientz-48 Tissuelyser (Jingxin, Shanghai, China) for high-throughput grinding. They were then sonicated at 200 W for 10 min using a Computer Numerical Control (CNC) ultrasonic cleaner (KQ5200DB, Jiangsu, China). After centrifuging at 10,000 r/min for 5 min, the supernatant was gathered and then passed through 0.22 μm filter films (Immobilon, Millipore, Germany). The Agilent 1290 UHPLC integrated with an electrospray ionization-time-of-flight mass spectrometer (ESI-TOF-MS) was utilized for LC-MS analysis. An Agilent ZORBAX Eclipse XDB-C18 column (1.8 μm, 3.0 mm × 150 mm) was operated for metabolite separation. The combination of eluents A (0.1% formic acid) and B (100% acetonitrile) produced a step gradient. The shikimic acid elution gradient was set at a flow rate of 0.4 mL/min and a column temperature of 30 °C: 0–1 min (0% B), 1–3 min (5% B), 3–5 min (90% B), 5–6 min (90% B) and 6–8 min (0% B). Calibration curves were constructed using shikimic acid standard substances with known concentrations, and based on these curves, the content of shikimic acid in the *L. reuteri* SXDT-32 supernatant and colonic chyme metabolites was determined.

### Cell lines and treatment

The human colorectal cancer cell line Caco-2 (ATCC HTB-37, RRID: CVCL_0025) was acquired from American Type Culture Collection (ATCC; Manassas, VA, USA). Cells were incubated in DMEM (Gibco, 11330-032) medium supplemented with 10% FBS (Gibco, 16000-044) in a humidified atmosphere containing 5% CO_2_ at 37 °C.

Prior to the formal experiment, the Cell Counting Kit-8 (Solarbio, CA1210) was employed to assess the impact of different concentrations of LPS (0 μg/mL, 1 μg/mL, 5 μg/mL, 10 μg/mL, 50 μg/mL, 100 μg/mL, 150 μg/mL) (Sigma, L2880), shikimic acid (SA) (0 mmol/L, 1 mmol/L, 5 mmol/L, 10 mmol/L, 20 mmol/L, 100 mmol/L) (Sigma, S5375) and *L. reuteri* SXDT-32 supernatant (0%, 1%, 2%, 4%, 6%, 8%, 10%, 12%) on the viability of Caco-2 cells after 12-h treatment to determine appropriate dosing regimens. During the determination of the LPS concentration, the level of IL-1β within the supernatant of Caco-2 cells exposed to different LPS concentrations was measured using an ELISA kit (BBI, D711047-0048). Subsequently, Caco-2 cells (1 × 10^5^ cells/mL) were randomly divided into six groups: PBS treatment (C), LPS (50 μg/mL) treatment (L), SA (20 mmol/L) plus LPS treatment (LS), *L. reuter*i SXDT-32 (1 × 10⁸ CFU/mL) plus LPS treatment (LR), *L. reuteri* SXDT-32 supernatant (2%) plus LPS treatment (LRS), and PI3K inhibitor: LY294002 (10 μmol/L) (Lablead, L1346) plus LPS treatment (LY) [27]. Cells were separately treated with PBS, SA, *L. reuteri* SXDT-32, *L. reuteri* SXDT-32 supernatant, or LY294002 for 12 h, followed by 12-h LPS treatment to induce inflammation. Finally, the cells were collected for Western blot analyses after twice washing with PBS.

### Statistical analysis

Values were expressed as mean ± SEM for both in vivo and in vitro trials. The Student's *t*-test was used to compare the two groups. One-way analysis of variance (ANOVA) was used to compare differences among multiple groups, and post hoc analysis was performed by Tukey’s multiple comparisons test. *P < 0.05, **P < 0.01 and ***P < 0.001 were considered acceptable levels of significance. All data plotting was performed using GraphPad Prism 10.

## Results

### *L. reuteri* SXDT-32 improved body growth and intestinal barrier function

**Fig. 1:**
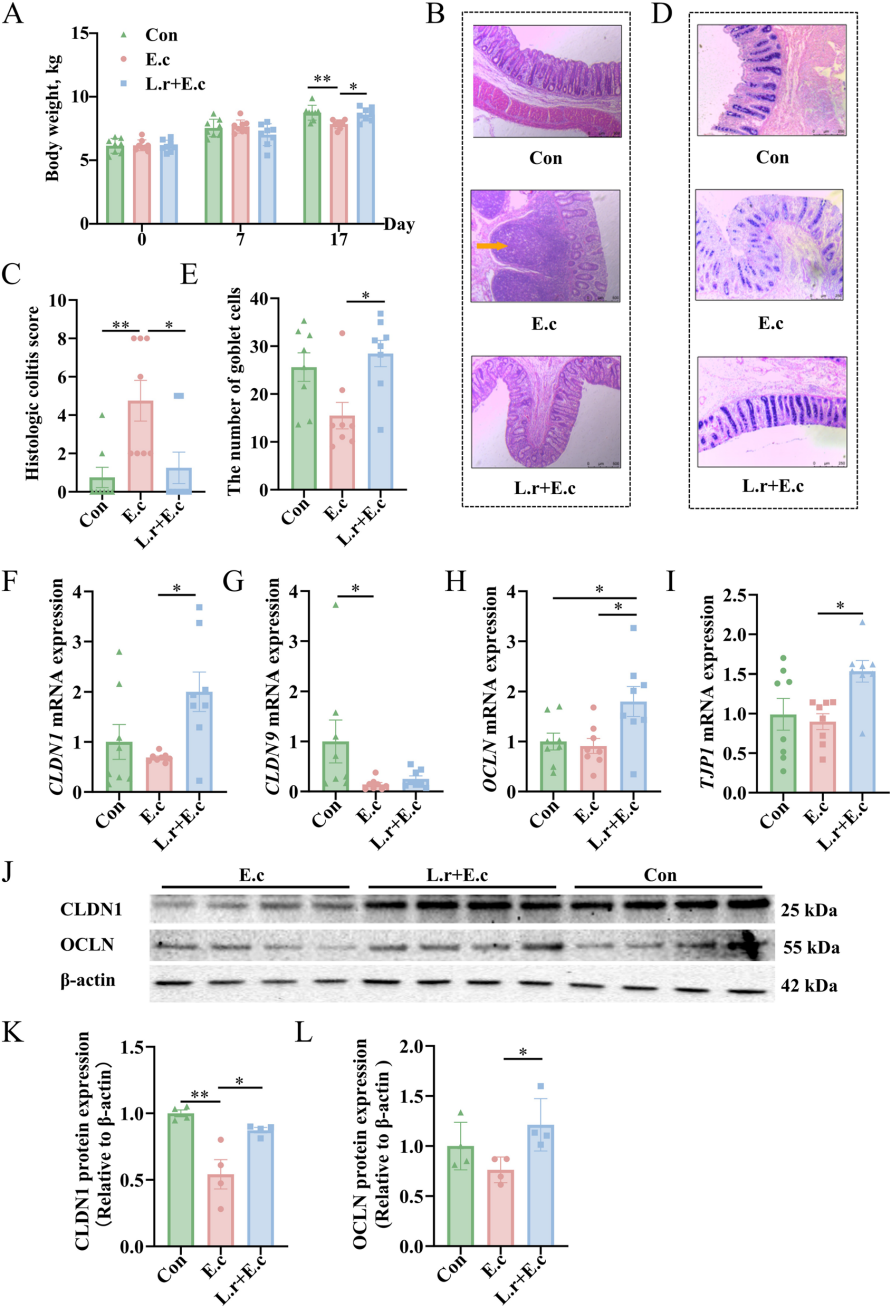
*L. reuteri* SXDT-32 improved body weight and intestinal barrier of piglets. **A** Body weight of piglets on d 0, d 7, and d 17. **B** H&E staining of colon tissue. Scale bar: 500 μm. **C** The histologic colitis score of piglets. **D** AB-PAS staining of colon tissue. Scale bar: 250 μm. **E** Goblet cells number in the colon of piglets. **F–I** *CLDN1*, *CLDN9*, *OCLN*, and *TJP1* mRNA levels in the colonic mucosa. **J**–**L** CLDN1 and OCLN protein levels in the colonic mucosa. *n* = 8 per group (**A**–**I**), *n* = 4 per group (**J–L**). Data are shown as mean ± SEM. Statistical significance was assessed via one-way ANOVA. *P < 0.05, **P < 0.01

The impacts of *L. reuteri* SXDT-32 on body weight and intestinal barrier after *E. coli*-induced colonic inflammation were evaluated. The results showed that weights did not differ significantly among groups on both d 0 and d 7 (*P* > 0.05; Fig. 1A). However, on d 17, the body weights of the Con and L.r + E.c groups were notably higher than those of the E.c group (*P* < 0.05; Fig. 1A). In addition, no apparent weight differences were observed between the Con and L.r + E.c groups (*P* > 0.05; Fig. 1A). Next, H&E staining of the colon revealed that the L.r + E.c group exhibited a considerably lower histological colitis score compared to the E.c group (*P* < 0.05; Fig. 1B and C). AB-PAS staining of the colon showed that the L.r + E.c group had more goblet cells than the E.c group (*P* < 0.05; Fig. 1D and E). Furthermore, the mRNA level of *CLDN9* (*P* < 0.05) was reduced in the E.c group relative to the Con group. Compared to the E.c group, *L. reuteri* SXDT-32 significantly upregulated the mRNA expression of *CLDN1*, *OCLN*, and *TJP1* (*P* < 0.05; Fig. 1F–I). Western blot analyses revealed that the level of CLDN1 protein was significantly reduced in the E.c group compared to the Con group (*P* < 0.01). After the addition of *L. reuteri* SXDT-32, the levels of both CLDN1 and OCLN proteins were significantly increased compared with those in the E.c group (*P* < 0.05; Fig. 1J–L).

### *L. reuteri* SXDT-32 alleviated *E. coli*-induced colonic inflammation with increased colon *L. reuteri* abundance

**Fig. 2:**
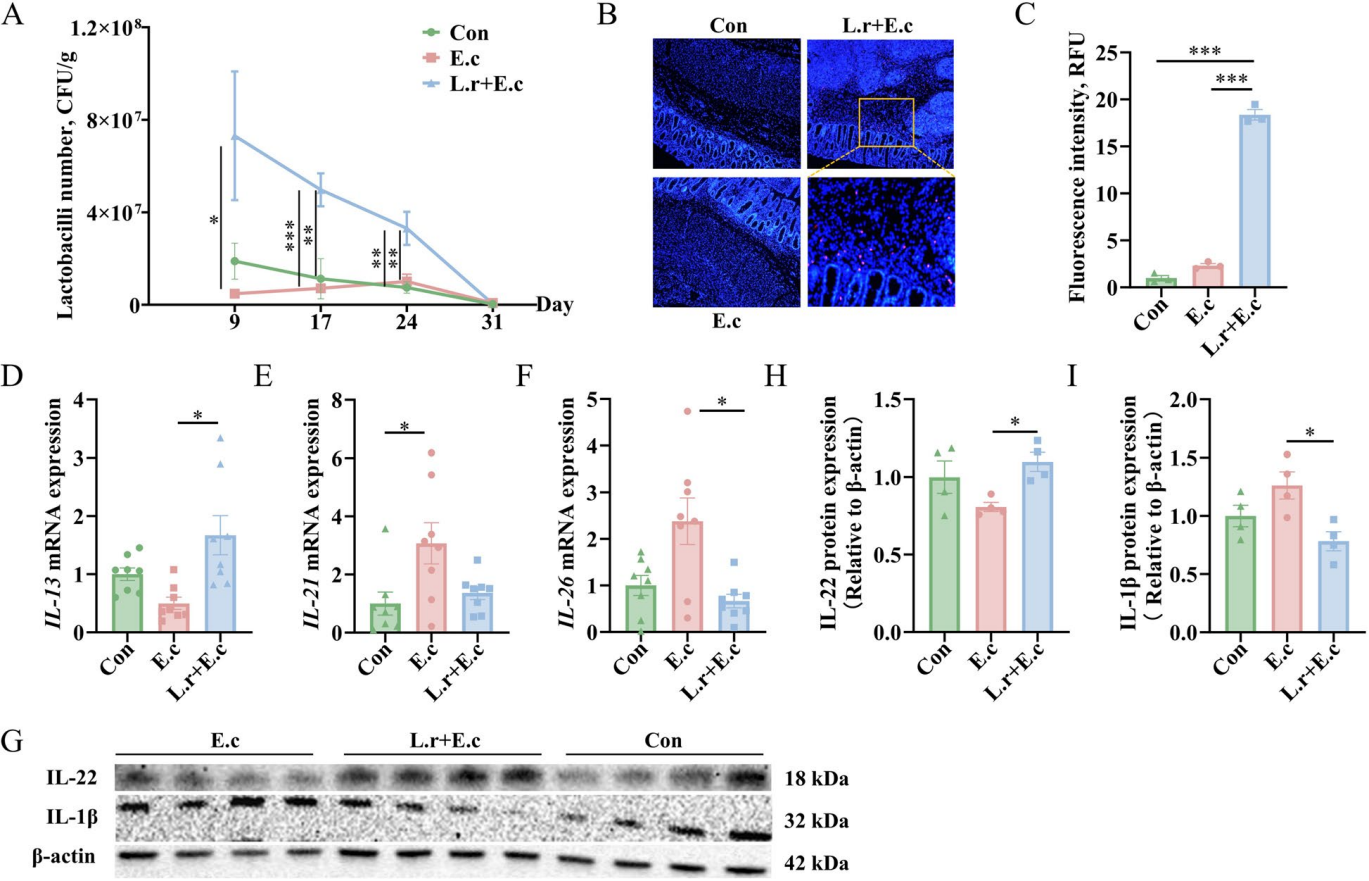
*L. reuteri* SXDT-32 alleviated *E. coli*-induced colonic inflammation with increased colon *L. reuteri* abundance. **A** The lactobacilli count in feces on d 9, d 17, d 24 and d 31. **B** and **C** Detection of the abundance of *L. reuteri* in colon by FISH and bar chart of *L. reuteri* fluorescence intensity. RFU: relative fluorescence units. *L. reuteri*: red. Scale bar: 250 μm. **D**–**F** *IL-13*, *IL-21* and *IL-26* mRNA levels in the colonic mucosa. **G**–**I** IL-22 and IL-1β protein levels in the colonic mucosa. *n* = 8 per group (**A**, **D–F**), *n* = 4 per group (**G**–**I**), *n* = 3 per group (**B** and **C**). Data are shown as mean ± SEM. Statistical significance was assessed via one-way ANOVA. *P < 0.05, **P < 0.01, ***P < 0.001

To clarify the abundance of *L. reuteri* in the colon and the impacts of *L. reuteri* SXDT-32 on colonic inflammation in piglets, the spread plate method and FISH were initially used to determine the abundance of *L. reuteri* in the colon. The spread plate method results indicated a significant increase in the count of lactobacilli in the feces of the L.r + E.c group compared with the other two groups on d 17 and d 24 of the experiment (*P* < 0.01; Fig. 2A). FISH revealed markedly stronger red fluorescence (from Cy3-labelled *L. reuteri*) in the L.r + E.c group compared to the other two groups (*P* < 0.001; Fig. 2B and C).

Subsequently, inflammatory factors in the colonic mucosa were assessed. The results showed that *IL-21* mRNA level was significantly increased in the E.c group compared to the Con group (*P* < 0.05). Compared with the E.c group, the L.r + E.c group showed a notably elevated *IL-13* mRNA expression (*P* < 0.05) but a significantly decreased *IL-26* mRNA level (*P* < 0.05; Fig. 2D–F). Moreover, Western blot revealed markedly increased IL-22 protein level and decreased IL-1β protein level in the L.r + E.c group relative to the E.c group (*P* < 0.05; Fig. 2G and H).

### *L. reuteri* SXDT-32 alleviated *E. coli*-induced intestinal inflammation by inhibiting PI3K-Akt signaling pathway

**Fig. 3:**
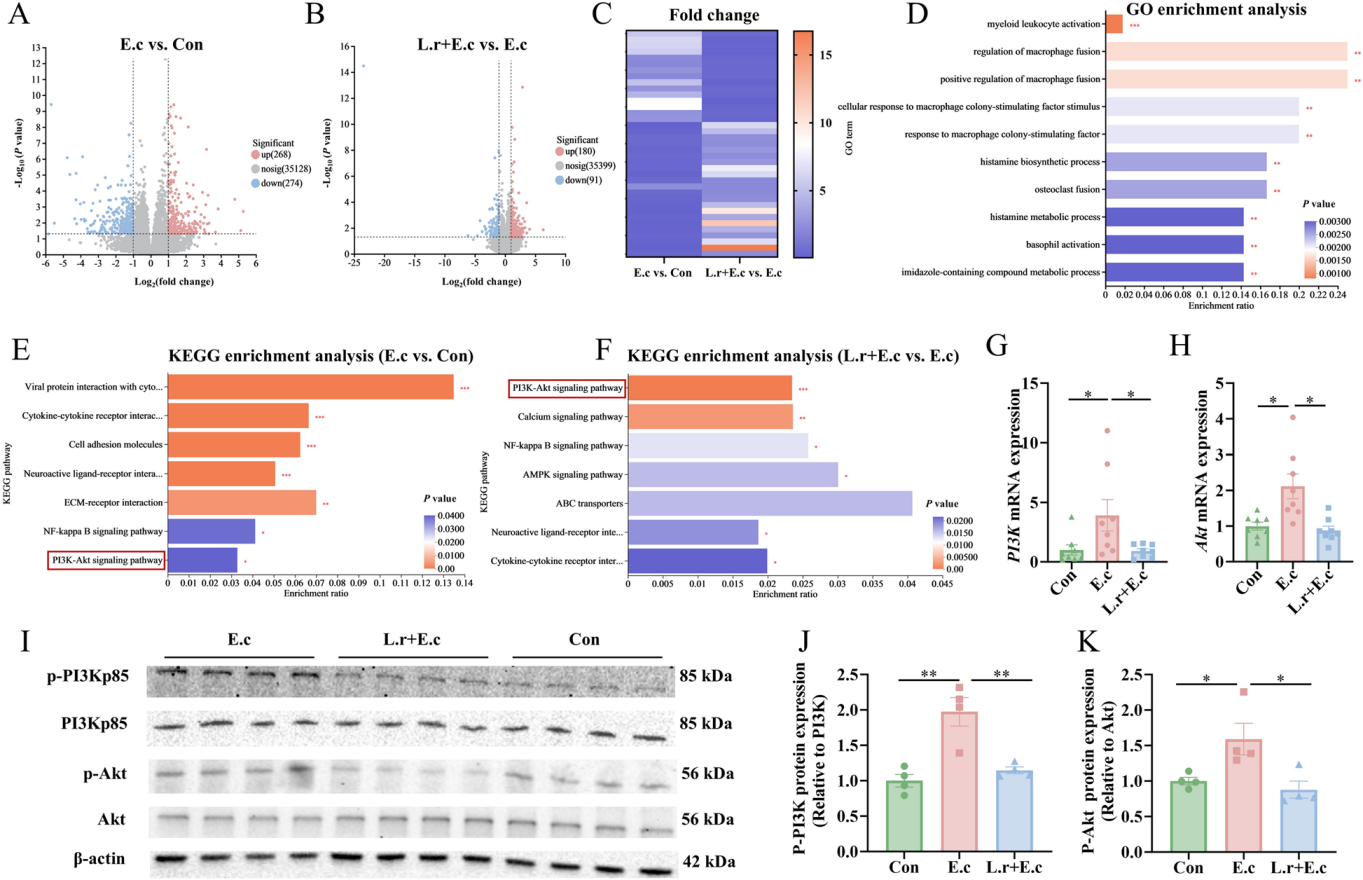
*L. reuteri* SXDT-32 relieved intestinal inflammation caused by *E. coli* by inhibiting PI3K-Akt signaling pathway. **A** and **B** Volcano plots for DEGs in E.c vs. Con and in L.r + E.c vs. E.c. **C** Fold change for DEGs in E.c vs. Con and L.r + E.c vs. E.c. **D** GO enrichment analysis of the 37 DEGs reversed by *L. reuteri* SXDT-32. **E** KEGG pathway analysis for DEGs in E.c vs. Con. **F** KEGG pathway analysis for DEGs in L.r + E.c vs. E.c. **G** and **H** *PI3K* and *Akt* mRNA levels in the colonic mucosa. **I**–**K** p-PI3K and p-Akt protein levels in the colonic mucosa. *n* = 8 per group (**A**–**H**), *n* = 4 per group (**I**–**K**). Data are shown as mean ± SEM. Statistical significance was assessed via one-way ANOVA. *P < 0.05, **P < 0.01, ***P < 0.001

To elucidate the detailed mechanism by which *L. reuteri* SXDT-32 alleviates intestinal damage caused by *E. coli*, RNA sequencing of the colonic mucosa was conducted. The data indicated that 268 upregulated and 274 downregulated differentially expressed genes (DEGs) were identified in E.c vs. Con (Fig. 3A), and 180 upregulated and 91 downregulated DEGs were detected in L.r + E.c vs. E.c (Fig. 3B). Interestingly, the *E. coli*-induced alterations of 37 DEGs were reversed by *L. reuteri* SXDT-32 supplementation (Fig. 3C). Gene Ontology (GO) functional enrichment found that the 37 DEGs were mainly associated with immune processes, including myeloid leukocyte activation, regulation of macrophage fusion, eosinophil activation, and negative regulation of humoral immune response, etc. (Fig. 3D). Additionally, the analysis of the Kyoto Encyclopedia of Genes and Genomes (KEGG) pathway analysis indicated that DEGs in E.c vs. Con and in L.r + E.c vs. E.c were both enriched in the PI3K-Akt pathway, with a particularly strong enrichment observed in the latter (Fig. 3E and F). These observations suggested that *L. reuteri* SXDT-32 had the ability to modulate the PI3K-Akt signaling pathway.

To confirm this hypothesis, we measured the mRNA and protein expression levels of pathway-related genes in the colonic mucosa of piglets. The results showed that compared with the Con group, *E. coli* significantly increased the levels of *PI3K* and *Akt* mRNA, and p-PI3K and p-Akt proteins (*P* < 0.05, Fig. 3 G–K). In contrast, supplementation with *L. reuteri* SXDT-32 significantly reduced these levels compared to the E.c group (*P* < 0.05, Fig. 3G–K). Hence, these experiments demonstrated that *L. reuteri* SXDT-32 could alleviate intestinal inflammation caused by *E. coli* through inhibition of the PI3K-Akt pathway.

### Identification of differentially expressed metabolites

**Fig. 4:**
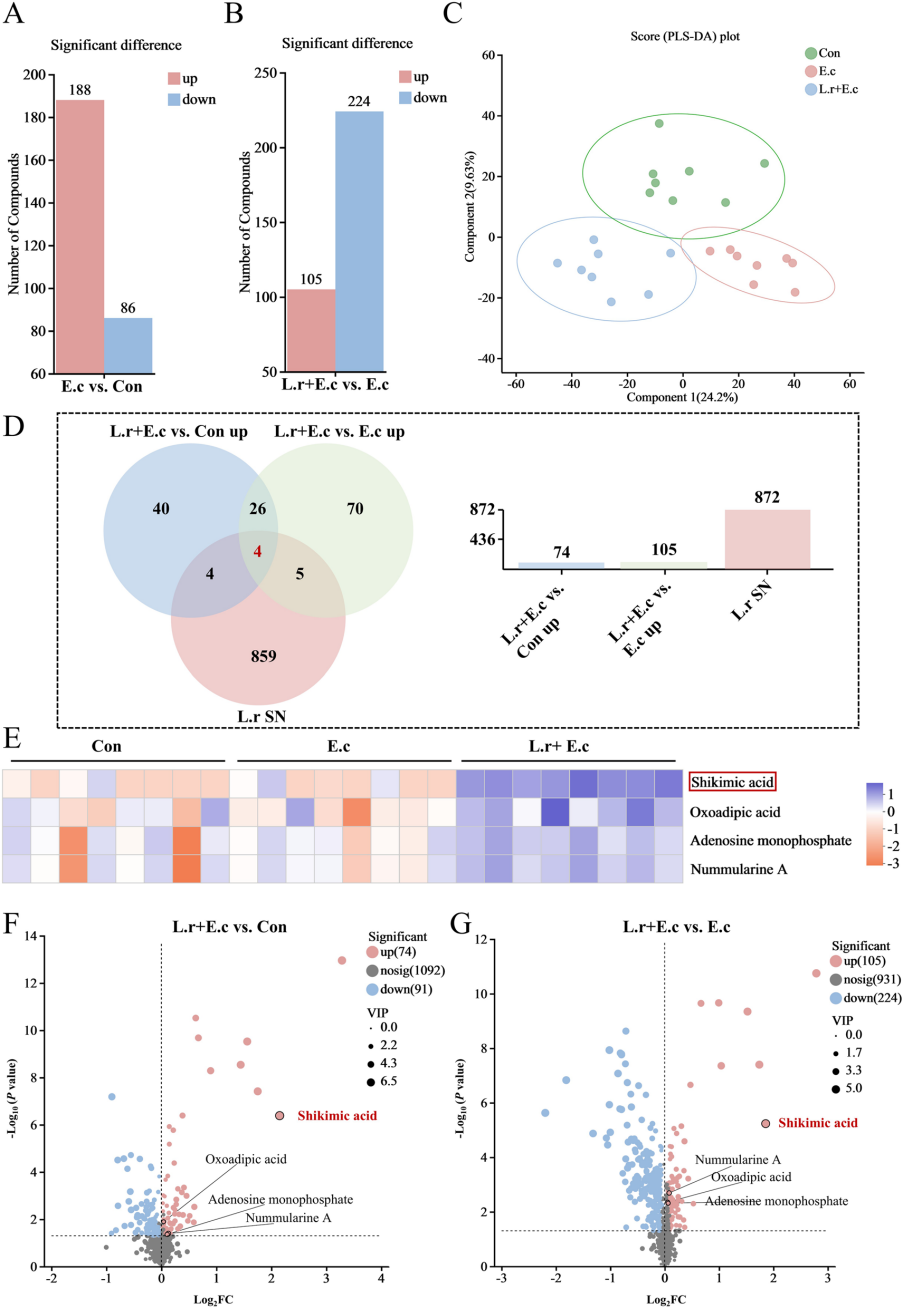
Identification of differentially expressed metabolites. **A** and **B** Histogram plots of differential metabolites in E.c vs. Con and in L.r + E.c vs. E.c. **C** PLS-DA score plot of metabolites in the Con, E.c, and L.r + E.c groups. **D** Venn diagram analysis for differential metabolites. L.r + E.c vs. Con up: Metabolites significantly increased in L.r + E.c vs. Con; L.r + E.c vs. E.c up: Metabolites significantly increased in L.r + E.c vs. E.c; L.r SN: Metabolites found in the supernatant of *L. reuteri* SXDT-32. **E** Heatmap of core differential metabolites. **F** and **G** Volcano plot for differentially enriched metabolites in L.r + E.c vs. Con and in L.r + E.c vs. E.c. *n* = 8 per group

Metabolites produced by bacteria play a crucial role in enabling beneficial bacteria to exert their beneficial properties [28]. To further investigate the key metabolites of *L. reuteri* SXDT-32 in alleviating *E. coli*-induced intestinal damage, we performed the non-targeted metabolomics assay using colonic mucosa and *L. reuteri* SXDT-32 supernatant, respectively. The data indicated that 274 differential metabolites were detected in E.c vs. Con, including 188 upregulated and 86 downregulated metabolites (Fig. 4A). 329 differential metabolites were detected in L.r + E.c vs. E.c, including 105 upregulated (such as shikimic acid, oxoadipic acid, adenosine monophosphate, nummularine A; see Table S3 for a full list) and 224 downregulated metabolites (Fig. 4B). Partial least squares discriminant analysis (PLS-DA) plots of the identified metabolites in the colonic mucosa revealed a clear separation among Con, E.c and L.r + E.c groups (Fig. 4C). Additionally, a total of four key differential metabolites were identified using Venn diagram analysis (Fig. 4D). The four metabolites were not only found in the *L. reuteri* SXDT-32 supernatant, but also at markedly higher levels in the L.r + E.c group compared to the other two groups. Among the four metabolites, SA exhibited a more significant response to *L. reuteri* SXDT-32, as indicated by a lower *P* value (Fig. 4E–G). These results suggested that SA may be a key metabolite produced by *L. reuteri* SXDT-32 and responsible for alleviating intestinal inflammation.

### *L. reuteri* SXDT-32 synthesized SA protects against intestinal inflammation

**Fig. 5:**
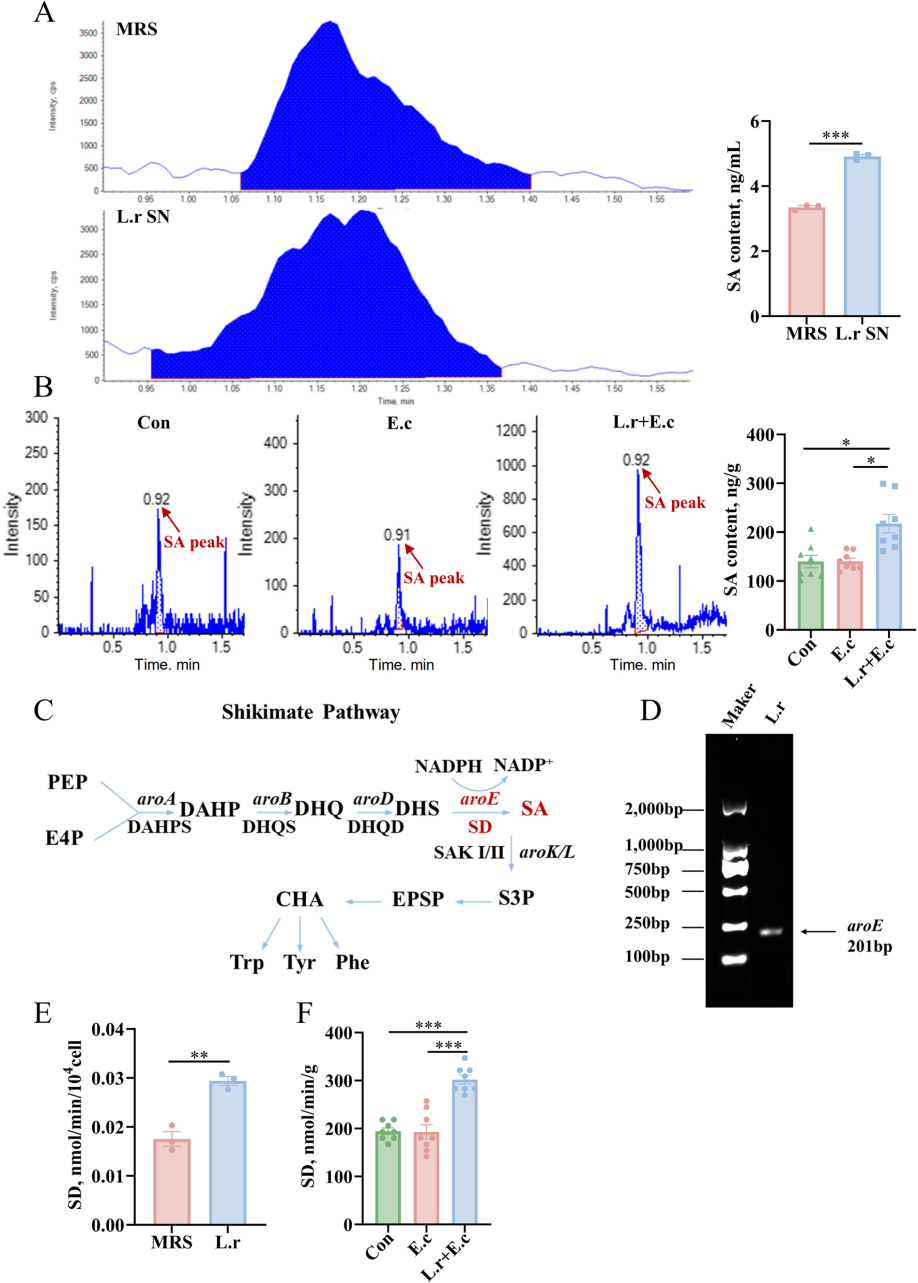
*L. reuteri* SXDT-32 synthesized SA. **A** The LC-MS detection peak diagrams for SA in MRS broth and *L. reuteri* SXDT-32 supernatant, and bar chart of SA content. L.r SN: *L. reuteri* SXDT-32 supernatant. **B** The LC-MS detection peak diagrams for SA in the colonic chyme, and bar chart of SA content. **C** Shikimate pathway. PEP: phosphoenolpyruvate; E4P: D-erythrose 4-phosphate; DAHPS: 3-deoxy-D-arabino-heptulosonate-7-phosphate synthase (encoded by *aroA*); DAHP: 3-deoxy-D arabino-heptulosonate-7-phosphate; DHQS: 3-dehydroquinate synthase (encoded by *aroB*); DHQ: 3-dehydroquinate; DHQD: 3-dehydroquinate dehydrogenase (encoded by *aroD*); DHS: 3-dehydroshikimate; SD: shikimate dehydrogenase (encoded by *aroE*); SAK I/II: shikimic Acid Kinase I and shikimic Acid Kinase II (encoded by *aroK* and *aroL* respectively); S3P: shikimate-3phosphate; EPSP: 5-enolpyruvylshikimate-3-phosphate; CHA: chorismate; Phe: Phenylalanine; Tyr: tyrosine; Trp: tryptophan. **D** The *aroE* gene in *L. reuteri* SXDT-32. L.r: *L. reuteri* SXDT-32. **E** The activity of SD in the *L. reuteri* SXDT-32. **F** The activity of SD in the colonic mucosa. *n* = 3 per group (**A** and **E**), *n* = 8 per group (**B** and **F**). Data are shown as mean ± SEM. Statistical significance was assessed via Student's *t*-test (**A** and **E**) or one-way ANOVA (**B** and **F**). *P < 0.05, **P < 0.01, ***P < 0.001

To identify whether SA can be directly synthesized by *L. reuteri* SXDT-32, we initially employed LC-MS to measure the level of SA in the supernatant of *L. reuteri* SXDT-32 and the colonic mucosa of piglets. The findings indicated that the content of SA in the supernatant of *L. reuteri* SXDT-32 was considerably higher than that in the MRS broth (*P* < 0.001; Fig. 5A). Consistently, the SA content in the colonic chyme of piglets was substantially higher in L.r + E.c group compared to the other two groups of piglets that were not fed *L. reuteri* SXDT-32 (*P* < 0.05; Fig. 5B). Notably, the key gene (*aroE*) for SA biosynthesis was found in the *L. reuteri* SXDT-32 (Fig. 5C and D), and higher activity of SD (encoded by *aroE*) was detected both in the *L. reuteri* SXDT-32 (*P* < 0.01; Fig. 5E) and in the colonic mucosa of piglets fed with *L. reuteri* SXDT-32 (*P* < 0.001; Fig. 5F). This indicated that SA was a metabolite produced by *L. reuteri* SXDT-32 that may play a role in protecting piglets from intestinal inflammation caused by *E. coli*.

### SA alleviated intestinal inflammation through inhibiting PI3K-Akt signaling pathway

**Fig. 6:**
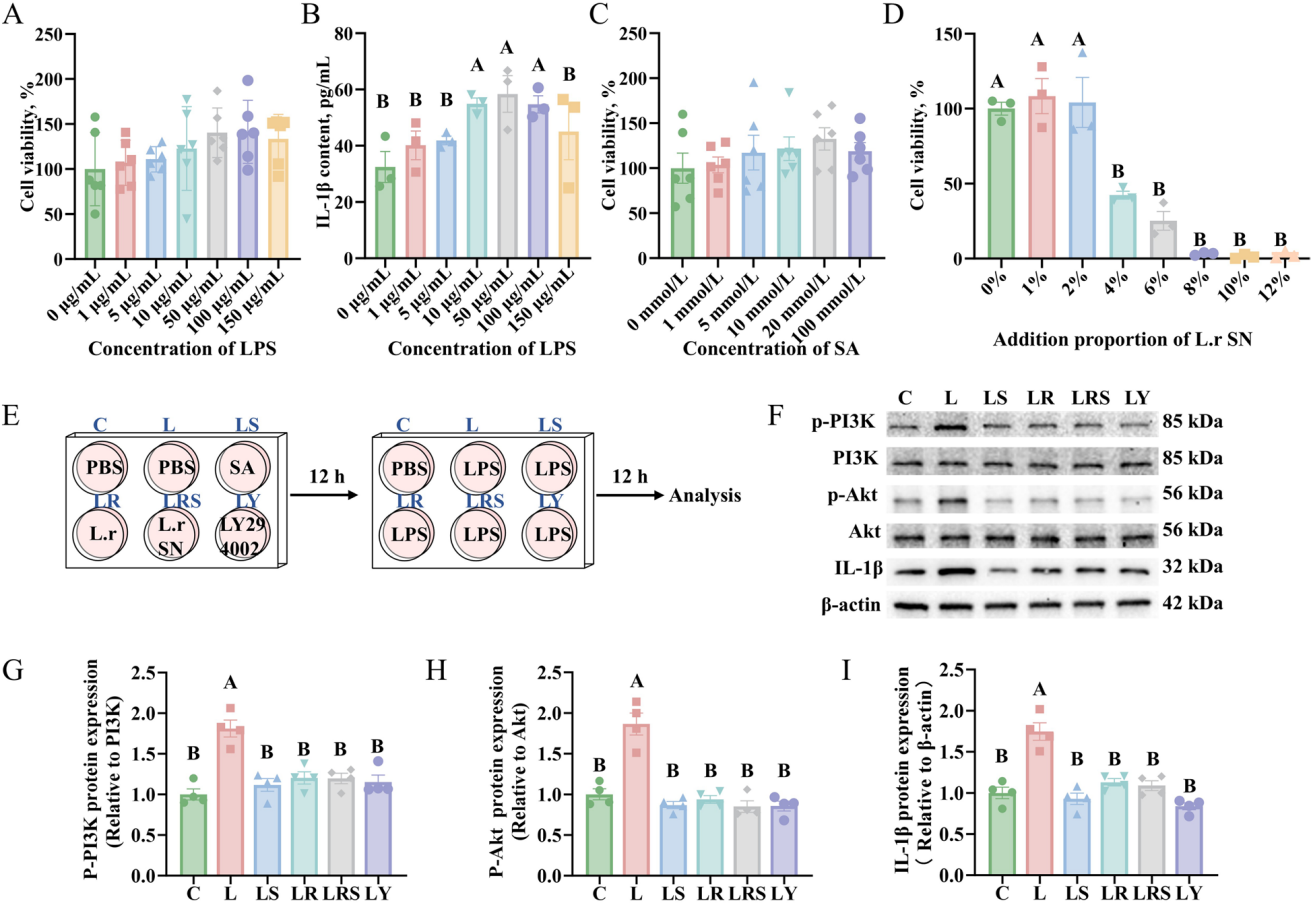
SA alleviated intestinal inflammation through suppression of the PI3K-Akt signaling pathway. **A** The impact of LPS on Caco-2 cell viability. **B** The impact of LPS on IL-1β content in Caco-2 cell supernatants. **C** The impact of SA on Caco-2 cell viability. **D** The impact of *L. reuteri* SXDT-32 supernatant on Caco-2 cell viability. L.r SN: *L. reuteri* SXDT-32 supernatant. 1%, 2%, 4%, 6%, 8%, 10%, 12%: L.r SN volume proportion in cell incubated medium. **E** Schematic diagram of drug administration in Caco-2 cells. PBS: Phosphate-Buffered Saline; L.r: *L. reuteri* SXDT-32; L.r SN: *L. reuteri* SXDT-32 supernatant; LY294002: PI3K inhibitor. **F**–**I** p-PI3K, p-Akt and IL-1β protein levels in Caco-2. *n* = 6 per group (**A** and **C**), *n* = 3 per group (**B** and **D**), *n* = 4 per group (**F**–**I**). Data are shown as mean ± SEM. Statistical significance was assessed via one-way ANOVA. Values with no common superscripts mean significant difference (*P* < 0.01)

To determine whether the SA produced by *L. reuteri* SXDT-32 can alleviate intestinal inflammation by inhibiting the PI3K-Akt signaling pathway, an in vitro inflammation model was established using LPS to stimulate Caco-2 cells. This model was then employed to evaluate the direct protective effect of SA treatment. Initially, the survival rate of Caco-2 cells was assessed following a 12-h exposure to varying concentrations of LPS, SA, and *L. reuteri* SXDT-32 supernatant. The findings showed that varying concentrations of LPS did not significantly affect the viability of Caco-2 cells (*P* > 0.05; Fig. 6A). However, the content of IL-1β reached its peak in the cell supernatants when the concentration of LPS was set at 50 μg/mL (*P* < 0.05; Fig. 6B). SA did not affect the viability of Caco-2 cells (*P* > 0.05; Fig. 6C). Moreover, the *L. reuteri* SXDT-32 supernatant had no obvious impact on cell viability when added at a proportion below 2% (Fig. 6D). Therefore, we selected 50 μg/mL LPS, 20 mmol/L SA, and 2% *L. reuteri* SXDT-32 supernatant for further experimentation.

Caco-2 cells were subjected to a 12-h pre-treatment with SA (20 mmol/L), *L. reuteri* SXDT-32 (1 × 10⁸ CFU/mL), *L. reuteri* SXDT-32 supernatant (2%), or LY294002 (10 μmol/L), followed by a 12-h stimulation with LPS (50 μg/mL) to induce inflammation (Fig. 6E). The cells were subsequently collected for the assessment of protein expression within the PI3K-Akt pathway and the levels of inflammatory cytokines. Western blot analyses illustrated that LPS treatment enhanced the protein expression levels of p-PI3K, p-Akt, and IL-1β in Caco-2 cells compared with the control group (*P* < 0.01; Fig. 6F–I). However, SA, *L. reuteri* SXDT-32, *L. reuteri* SXDT-32 supernatant, and LY294002 attenuated the increases in the expression levels of these three proteins that were induced by LPS treatment (*P* < 0.01; Fig. 6F–I), and no substantial variations were observed among the four treatments (*P* > 0.05). Overall, these results suggested that SA could alleviate intestinal inflammation through suppression of the PI3 K-Akt pathway, and the effect of SA was similar to that of *L. reuteri* SXDT-32, *L. reuteri* SXDT-32 supernatant and LY294002.

## Discussion

Colitis caused by bacterial infection is a grave and escalating global health issue [1]. Reducing inflammation and maintaining barrier homeostasis are proposed as the main treatment strategies. In our study, weaned piglets injected intraperitoneally with *E. coli* showed significant weight loss, colon damage (increased histological colitis scores, decreased goblet cell numbers), elevated pro-inflammatory cytokines, reduced anti-inflammatory cytokines, and decreased tight junction protein expression in the colon, resulting in serious intestinal injury in piglets. In contrast, *L. reuteri* SXDT-32 supplementation mitigated *E. coli*-induced intestinal damage, emphasizing the critical role of *L. reuteri* SXDT-32 in intestinal inflammation. *L. reuteri*, a common beneficial bacterium that is easy to obtain and maintain, has been reported to enhance jejunal architecture in piglets, which is demonstrated by increased villus length and diminished crypt depth, and to improve intestinal barrier function [28–30]. Consistently, *L. reuteri* has been shown to reduce pro-inflammatory cytokines, increase anti-inflammatory cytokines [31], and protect against pathogenic infections in inflammatory bowel disease (IBD) [32]. These results confirmed the beneficial roles of *L. reuteri* SXDT-32 in reducing intestinal inflammation.

Although this study confirmed that *L. reuteri* SXDT-32 can alleviate intestinal inflammation, its mechanism of operation still remains unknown. We hypothesized that the protective benefits of *L. reuteri* SXDT-32 could be achieved by altering host cell signaling pathways. Then, we screened for signaling pathways that might be affected by *L. reuteri* SXDT-32 using transcriptomic analysis. The results showed that DEGs in E.c vs. Con and L.r + E.c vs. E.c were both significantly enriched in PI3K-Akt pathway. A large number of studies indicated that PI3K-Akt pathway is intimately linked to cellular multiplication, differentiation, migration, and programmed demise and occupies a crucial position in intestinal inflammation [33, 34]. For example, a recent investigation has illustrated that the triggering of the PI3K-Akt pathway participates in ulcerative colitis development [35]. Moreover, another study has found that inhibiting the phosphorylation of proteins within the PI3K-Akt signaling pathway can exert a potent anti-inflammatory impact by decreasing pro-inflammatory cytokines [36]. These evidences suggest that inhibiting PI3K-Akt signaling can represent a novel therapeutic strategy for intestinal inflammation. In our study, *E. coli* increased the mRNA levels of *PI3K* and *Akt*, as well as the protein levels of p-PI3K and p-Akt in the colonic mucosa of piglets, indicating activation of the PI3K-Akt signaling pathway in the E.c group. Instead, compared with the E.c group, *PI3K* and *Akt* mRNA levels, and p-PI3K and p-Akt protein levels were reduced following *L. reuteri* SXDT-32 administration. And after *L. reuteri* SXDT-32 treatment, IL-1β protein level was noticeably declined while IL-22 protein level significantly increased. Consequently, the outcomes suggested that *L. reuteri* SXDT-32 can inhibit *E. coli*-induced intestinal inflammation by inhibiting the PI3K-Akt signaling pathway. Supportive evidence came from Dong's report, which showed that triggering of the PI3K-Akt results in significantly upregulated *IL-6*, *IL-8*, *IL-1β*, and *TNF-α* mRNA levels, but decreased *IL-10* mRNA level. However, inhibition of this pathway is capable of relieving ulcerative colitis caused by DSS [37]. Dai et al. also observed that application of VSL#3 (a mixture of *Lactobacillus acidophilus*, *Lactobacillus plantarum*, *Lactobacillus casei*, *Lactobacillus delbrueckii* subspecies *bulgaricus*, *Bifidobacterium breve*, *Bifidobacterium longum*, *Bifidobacterium infantis* and *Streptococcus salivarius* subspecies *thermophilus*) beneficial bacteria alleviates various ulcerative colitis symptoms in mice, including stunted growth, diarrhea, and fecal bleeding, while also inhibiting the PI3K-Akt pathway [38]. These studies showed that *L. reuteri* SXDT-32 has a material basis for relieving intestinal inflammation by inhibiting the PI3K-Akt pathway.

Metabolites produced by gut-derived microbes have a significant impact on microbe-host interactions [39]. For instance, it has been demonstrated that beneficial bacteria can strengthen the function of the intestinal barrier and modulate the immune system via the production of diverse metabolites, such as short-chain fatty acids, bacteriocins, reuterin [40, 41]. In our study, we found that SA was significantly increased in the colonic mucosa and colonic chyme of *L. reuteri* SXDT-32-fed piglets. Consistently, a considerable quantity of SA was identified in the *L. reuteri* SXDT-32 supernatant. SA, a natural organic acid with a six-membered carbon ring, possesses multiple biological features, including anti-hyperglycemic, anticancer, antimicrobial, anti-phlogistic, antioxidative, and anti-thrombogenic impacts [42]. For example, SA administration by gavage could alleviate DSS-induced colitis in mice and enhance intestinal immunity [43]. The mechanisms of SA action include scavenging of superoxide and hydroxyl radicals [44], activation of the Akt/Nrf2 signaling pathway [45], regulation of autophagy-related proteins and autophagic flux, and interference with the NF-κB and MAPK signaling pathways [46]. Therefore, we hypothesized that *L. reuteri* SXDT-32 produced SA, which played a key role in alleviating intestinal inflammation. It is well known that SA is synthesized via the shikimate pathway, a biosynthetic process found exclusively in plants, fungi, and microorganisms (Fig. 5C) [47]. Based on the pathway, studies found that *aroE* is a crucial gene in the synthesis of SA. The *aroE* gene encodes SD, an enzyme that directly catalyzes the conversion of 3-dehydroshikimate (DHS) to SA [48]. It's worth noting that the *aroE* gene was detected in the *L. reuteri* SXDT-32. Consistent with this, elevated SD was also detected in both the *L. reuteri* SXDT-32 and the colonic mucosa of piglets fed *L. reuteri* SXDT-32. Thus, we confirmed that *L. reuteri* SXDT-32 produced SA, which may be a key metabolite in alleviating intestinal inflammation.

Based on our findings, we hypothesized that SA also alleviated intestinal inflammation through inhibition of the PI3K-Akt signaling pathway, as we confirmed that *L. reuteri* SXDT-32 participated in the above process. We first determined that SA administration reversed LPS-induced upregulation of the protein levels of IL-1β, p-PI3K and p-Akt in Caco-2 cells. These data indicated that SA administration could alleviate intestinal inflammation by inhibiting the PI3K-Akt pathway. Similar research reported by Li et al. showed that SA reduces pro-inflammatory cytokines by suppressing the NF-κB and MAPK pathways, thereby ameliorating ulcerative colitis caused by DSS [43]. To further validate the suppressive effect of SA on the PI3K-Akt pathway, we compared the impact of SA with that of LY294002, which specifically blocks the activity of the PI3K enzyme, thereby inhibiting the PI3K-Akt signaling pathway [49]. The results indicated that SA had a comparable inhibitory effect on the PI3K-Akt pathway compared to PI3K inhibitors. There were no significant differences in suppression of the PI3K-Akt pathway and reduction of pro-inflammatory cytokine among treatments with SA, *L. reuteri* SXDT-32, and *L. reuteri* SXDT-32 supernatant. The effect might be attributed to shikimic acid produced by *L. reuteri* SXDT-32. Additionally, other metabolites produced by *L. reuteri* SXDT-32 may act in concert with shikimic acid to inhibit the PI3K-Akt pathway and decrease the levels of pro-inflammatory factors.

## Conclusion

Our research shows that pretreatment with *L. reuteri* SXDT-32 can improve intestinal barrier function and alleviate *E. coli*-induced intestinal inflammation in weaned piglets. Furthermore, *L. reuteri* SXDT-32 has the potential to synthesize SA. SA may act as a key regulatory factor in alleviating inflammation, and its mechanism involves the inhibition of the PI3K-Akt signaling pathway.

## Supplementary Information

Supplementary Material 1: Tables S1 Composition and nutrient level of basal diet. Tables S2 Primers employed in qPCR. Tables S3 L.r + E.c vs. E.c differential metabolites

## Data Availability

All data that support the results of this study are available from the corresponding author on reasonable request.

## Abbreviations

AB-PAS: Alcian Blue-Periodic Acid-Schiff
Akt: V-akt murine thymoma viral oncogene homolog
ACTB: Actin beta
CLDN1: Claudin 1
CLDN9: Claudin 9
DEGs: Differentially expressed genes
DLY: Duroc × Landrace × Yorkshire
FISH: Fluorescence in situ hybridization
GO: Gene Ontology
HE: Hematoxylin-eosin
IL-1β: Interleukin-1β
IL-13: Interleukin-13
IL-21: Interleukin-21
IL-22: Interleukin-22
IL-26: Interleukin-26
KEGG: Kyoto Encyclopedia of Genes and Genomes
LC-MS: Liquid Chromatography-Mass Spectrometer
LPS: Lipopolysaccharide
MRS: De Man, Rogosa and Sharpe
OCLN: Occludin
PBS: Phosphate-Buffered Saline
PI3K: Phosphatidylinositol 3-kinase
qRT-PCR: Quantitative real-time polymerase chain reaction
SA: Shikimic acid
SD: Shikimate dehydrogenase
VIP: Variable Importance in the Projection
