# Supplementary material for: Limosilactobacillus reuteri SXDT-32-derived shikimic acid protects against colonic inflammation in piglets by inhibiting the PI3K-Akt pathway
Source: J Anim Sci Biotechnol. 2025 Jun 13;16:84. doi: 10.1186/s40104-025-01221-w (PMC12164073; doi:10.1186/s40104-025-01221-w)
Supplement: Supplementary file 1 — Supplementary Material 1: Tables S1 Composition and nutrient level of basal diet. Tables S2 Primers employed in qPCR. Tables S3 L.r + E.c vs. E.c differential metabolites [file 40104_2025_1221_MOESM1_ESM.docx]

**Table S1** Composition and nutrient level of basal diet

| **Ingredient** | **%** | **Nutrient level** |  |
| --- | --- | --- | --- |
| Suckling corn | 29.48 | Digestible Energyb, MJ/kg | 14.4 |
| Extruded corn | 15 | Crude protein, % | 17.43 |
| Crushed rice | 15 | Calcium, % | 0.78 |
| Soybean meal 46% | 4.5 | Available Phosphorus, % | 0.54 |
| Whey powder | 8 | Lysine, % | 1.3 |
| Extruded soybean | 13 | Methionine, % | 0.48 |
| Fish meal | 3 | Methionine and Cystine, % | 0.77 |
| Wheat hydrolyzed protein | 3 | Threonine, % | 0.81 |
| Glucose | 4 | Tryptophan, % | 0.24 |
| Soya-bean oil | 1.5 |  |  |
| Calcium formate | 0.8 |  |  |
| Calcium bis | 0.5 |  |  |
| Lysine 98.5% | 0.65 |  |  |
| Methionine 98.5% | 0.16 |  |  |
| Threonine | 0.24 |  |  |
| Antioxidant | 0.1 |  |  |
| Tryptophan | 0.07 |  |  |
| Premixa | 1 |  |  |
| Total | 100 |  |  |

^a^ Supplied for each kilogram of the diet: vitamin A, 15,000 IU; vitamin D_3_, 4,500 lU; vitamin E, 72.5 mg; vitamin K_3_, 4.5 mg; vitamin B_1_, 4.32 mg; vitamin B_2_, 12 mg; vitamin B_6_, 4.86 mg; vitamin B_12_, 30 μg; biotin, 480 μg; folic acid, 1.764 mg; Calcium Pantothenate, 19.32 mg; nicotinamide, 41.58 mg; Fe, 165 mg; Cu, 110 mg; Mn, 60 mg; Zn, 80 mg; I, 0.8 mg; Se, 0.30 mg; Co, 0.6 mg

^b^ Calculated value

**Table S2** Primers employed in qPCR

| **Gene** | **Accession number** | **Primer sequences (5'→3')** |
| --- | --- | --- |
| *ACTB* | NM_007393.5 | F:GCGTAGCATTTGCTGCATGA |
|  |  | R:GCGTGTGTGTAACTAGGGGT |
| *OCLN* | NM_001163647.2 | F:CAGGTGCACCCTCCAGATTG |
|  |  | R:TATGTCGTTGCTGGGTGCAT |
| *CLDN1* | NM_001244539.1 | F:ATGCCTCCTCCCCTTTC |
|  |  | R:GCTTCTCGTTCACTTTCCC |
| *CLDN9* | NM_001161647.1 | F:GCACGCCATCATCCAGGACTTC |
|  |  | R:CCGCCCAGCCGAGGTAGAG |
| *TJP1* | XM_003480423.4 | F:CTCCAGGCCCTTACCTTTCG |
|  |  | R:GGGGTAGGGGTCCTTCCTAT |
| *IL-13* | NM_213803.1 | F:TGTGCCGCCCTGGAATCCC |
|  |  | R:TTGGTGTCTCGGATGTGCTTGC |
| *IL-21* | NM_214415.1 | F:GGTCATCTTCTCAGGCACA |
|  |  | R:CAATTCAGGGTCCAAGTCA |
| *IL-26* | NC_010447.5 | F:CGCTGTATTTCCTGTGCTT |
|  |  | R:TCCACTTGGTTTCTTGCAT |
| *PI3K* | XM_021102203.1 | F:CTCACTCAGGGAACTCAGAGC |
|  |  | R:GTTTCCTGGGAGGAATCCGT |
| *AKT* | XM_021081500.1 | F:TCAAGAACGACGGCACCTTCATC |
|  |  | R:CGCCACGGAGAAGTTGTTGAGG |

**Table S3** L.r+E.c vs. E.c differential metabolites

| **Metabolite** | **Regulate** | **E.c** | **L.r+E.c** |
| --- | --- | --- | --- |
| Monomethyl succinate | up | 4.27±0.08 | 4.58±0.06 |
| 2-hydroxyisoflavanone naringenin | up | 5.39±0.13 | 5.93±0.14 |
| 1,2-Epithiopropane | up | 5.25±0.05 | 5.54±0.05 |
| S-Hydroxymethylglutathione | up | 4.36±0.09 | 4.71±0.05 |
| 1,3,5-Triazin-2(1H)-one, 4-amino-1-(2-deoxy-beta-D-erythro-pentofuranosyl)- | up | 4.05±0.12 | 4.69±0.06 |
| N-Acetylcadaverine | up | 2.31±0.1 | 3.33±0.29 |
| 1-[3,4-Dihydroxy-5-(hydroxymethyl)-2-oxolanyl]-1,2,4-triazole-3-carboxamide | up | 2.67±0.05 | 3.24±0.23 |
| 6-Acetamidohexanoic acid | up | 2.72±0.11 | 3.48±0.12 |
| Dihydroergocryptine | up | 2.44±0.01 | 4.86±0.15 |
| Naepaine | up | 3.9±0.1 | 4.83±0.09 |
| Frangulanine | up | 4.61±0.02 | 5.19±0.08 |
| Viloxazine | up | 4.71±0.11 | 5.14±0.08 |
| PG(20:2(11Z,14Z)/20:3(6,8,11)-OH(5)) | up | 1.84±0.19 | 5.29±0.13 |
| Palmitoylcarnitine | up | 5.5±0.09 | 5.85±0.13 |
| N-Arachidonoylglycine | up | 4.62±0.12 | 4.98±0.1 |
| Cytosine | up | 3.71±0.11 | 4.38±0.1 |
| L-Hexahydro-3-imino-1,2,4-oxadiazepine-3-carboxylic acid | up | 4.46±0.04 | 4.69±0.05 |
| Suberic acid | up | 4.35±0.12 | 4.68±0.08 |
| SM(d16:1/20:3(8Z,11Z,14Z)-2OH(5,6)) | up | 3.3±0.11 | 4.59±0.09 |
| Solacauline | up | 0.67±0.15 | 4.67±0.15 |
| SM(d16:1/PGJ2) | up | 3.14±0.01 | 4.99±0.12 |
| Fluocortolone caproate | up | 4.84±0.1 | 5.28±0.1 |
| Nummularine A | up | 5.26±0.08 | 5.57±0.03 |
| All-trans-Hexaprenyl diphosphate | up | 2.6±0.22 | 5.35±0.13 |
| Melevodopa | up | 5.39±0.04 | 5.7±0.12 |
| Cholylglutamic acid | up | 4.73±0.03 | 5.17±0.07 |
| H-D-Val-Leu-Lys-pNA | up | 4.18±0.05 | 4.86±0.09 |
| Motolimod | up | 4.92±0.03 | 5.23±0.07 |
| Lidocaine | up | 1.55±0.01 | 5.17±0.34 |
| N-(5-acetamidopentyl)acetamide | up | 4.1±0.17 | 4.86±0.18 |
| Hydroxypropionylcarnitine | up | 2.96±0.16 | 3.69±0.14 |
| Tyrosyl-Glutamate | up | 5.44±0.06 | 5.7±0.04 |
| Tuliposide B | up | 5.15±0.09 | 5.64±0.06 |
| 7-Aminomethyl-7-carbaguanine | up | 4.73±0.1 | 5.25±0.06 |
| 6-[3-(carboxymethyl)phenoxy]-3,4,5-trihydroxyoxane-2-carboxylic acid | up | 3.29±0.13 | 3.76±0.1 |
| Arginylglutamic acid | up | 4.37±0.08 | 4.75±0.06 |
| 2-Methylguanosine | up | 5.79±0.04 | 6.02±0.04 |
| DIDP | up | 3.87±0.3 | 4.93±0.28 |
| Wyerone | up | 3.98±0.23 | 4.61±0.13 |
| Homocysteinesulfinic acid | up | 3.33±0.08 | 4.49±0.25 |
| Cis-4-Hydroxyproline | up | 4.66±0.03 | 4.99±0.06 |
| (13Z)-3-Hydroxyicos-13-enoylcarnitine | up | 4.36±0.08 | 5.26±0.18 |
| N-Acetylglutamine | up | 4.73±0.04 | 5.06±0.04 |
| Adenosine monophosphate | up | 6.09±0.08 | 6.36±0.03 |
| Methylnoradrenaline | up | 4.67±0.1 | 5.01±0.09 |
| 5,6-Dihydroxyprostaglandin F1a | up | 6.09±0.06 | 6.34±0.06 |
| Glyceric Acid | up | 4.94±0.05 | 5.17±0.04 |
| Cytidine 2'-phosphate | up | 5.1±0.07 | 5.4±0.04 |
| D-Ribulose 5-Phosphate | up | 4.33±0.1 | 4.77±0.06 |
| Citric Acid | up | 6.34±0.09 | 6.69±0.08 |
| 5-Hydroxymethyl-2'-deoxyuridine | up | 4.65±0.17 | 5.33±0.13 |
| L-4-Hydroxyglutamate semialdehyde | up | 6.65±0.06 | 6.95±0.07 |
| (S)-alpha-Amino-4-carboxy-3-furanpropanoic acid | up | 3.92±0.12 | 4.54±0.08 |
| 8-Hydroxyguanosine | up | 4.64±0.1 | 5.14±0.06 |
| Thymidine 3',5'-cyclic monophosphate | up | 5.55±0.14 | 6.05±0.16 |
| 3'-Adenylic Acid | up | 6.39±0.08 | 6.67±0.03 |
| Shikimic acid | up | 1.48±0.54 | 5.37±0.13 |
| N4-Acetylcytidine | up | 4.88±0.04 | 5.08±0.03 |
| Phenylacetylglycine | up | 5.46±0.04 | 5.79±0.13 |
| P-Tolyl Sulfate | up | 5.65±0.04 | 5.88±0.05 |
| (6b,7b,13R)-6,7-Diacetoxy-8,14-labdadiene-13-ol | up | 4.98±0.14 | 5.47±0.11 |
| Docosapentaenoic acid (22n-3) | up | 5.71±0.05 | 6.01±0.06 |
| N-Palmitoyl Valine | up | 4.24±0.09 | 4.67±0.09 |
| Docosanedioic acid | up | 3.82±0.13 | 4.45±0.14 |
| Tetracosanedioic acid | up | 4.73±0.1 | 5.25±0.11 |
| 5'-Guanylic Acid | up | 5.77±0.08 | 6.31±0.05 |
| Oxoadipic acid | up | 5.79±0.06 | 6.01±0.03 |
| 22-Hydroxydocosanoic acid | up | 4.43±0.08 | 4.75±0.1 |
| Laurocapram | up | 4.39±0.12 | 4.82±0.08 |
| N-Stearoyl Cysteine | up | 3.27±0.29 | 4.22±0.29 |
| N-Palmitoyl Glycine | up | 4.24±0.15 | 5.02±0.11 |
| Gamma-Glutamyltyrosine | up | 5.29±0.06 | 5.51±0.05 |
| 1-(2,6,6-Trimethyl-2-cyclohexen-1-yl)-1,6-heptadien-3-one | up | 4.48±0.15 | 5.1±0.11 |
| Maleic Acid | up | 6.18±0.04 | 6.38±0.03 |
| 2,4-Dihydroxypteridine | up | 5.22±0.07 | 5.5±0.04 |
| N-lactoyl-phenylalanine | up | 5.4±0.09 | 5.66±0.07 |
| N6-(Delta2-Isopentenyl)-adenine | up | 5.62±0.08 | 5.87±0.07 |
| Ketoleucine | up | 5.16±0.06 | 5.69±0.15 |
| Indican | up | 4.5±0.16 | 4.98±0.07 |
| 3-Methylcrotonylglycine | up | 3.62±0.04 | 3.94±0.08 |
| Imazethapyr | up | 5.13±0.08 | 5.53±0.05 |
| 2,3-Dihydroxycarbamazepine | up | 4.88±0.05 | 5.25±0.05 |
| 1-Methylguanosine | up | 5.68±0.05 | 5.95±0.05 |
| Xanthosine | up | 5.98±0.08 | 6.26±0.06 |
| (-)-Abscisic acid | up | 5.33±0.06 | 5.59±0.09 |
| Gamma-Glutamylleucine | up | 6.05±0.05 | 6.3±0.05 |
| Isorhamnetin 3-glucoside 7-rhamnoside | up | 3.82±0.15 | 4.92±0.1 |
| 5-Hydroxy-L-tryptophan | up | 4.33±0.09 | 4.66±0.1 |
| Trifluoroquinolone | up | 4.28±0.26 | 5.19±0.21 |
| Pyroglutamic Acid | up | 4.22±0.16 | 5.02±0.15 |
| Hydroxydeoxyguanosine | up | 5.55±0.08 | 5.92±0.09 |
| 2-Furoic Acid | up | 4.86±0.07 | 5.14±0.08 |
| Uridine 2',3'-cyclic phosphate | up | 4.62±0.05 | 4.85±0.06 |
| Cytidine 3'-Phosphate | up | 5.89±0.06 | 6.18±0.04 |
| Cytidine 3'-monophosphate | up | 5.54±0.06 | 5.79±0.03 |
| O-Phospho-4-hydroxy-L-threonine | up | 4.43±0.23 | 5.27±0.2 |
| Carbamoyl (2R)-2,5-diaminopentanoate | up | 4.95±0.03 | 5.16±0.06 |
| 1-(2-Hydroxyethoxy)methyl-5-methyluracil | up | 3.69±0.16 | 4.39±0.12 |
| 15-keto-Prostaglandin E2 | up | 5.7±0.05 | 6.01±0.08 |
| Aspartylhydroxyproline | up | 4.39±0.06 | 4.65±0.05 |
| Ethyl brevifolincarboxylate | up | 5.61±0.06 | 5.9±0.08 |
| 3-Methyl-2-Oxovaleric Acid | up | 5.45±0.07 | 5.97±0.15 |
| D-Galactose 1-Phosphate | up | 4.63±0.08 | 5.01±0.1 |
| D-Sedoheptulose 7-phosphate | up | 6.07±0.04 | 6.28±0.05 |
| Isocitrate | up | 5.91±0.06 | 6.14±0.03 |

The expression levels of metabolites are shown as mean ± SEM
